# A Rare Case Report of Extensive Polyostotic Gorham's Disappearing Bone Disease Involving the Upper Extremity

**DOI:** 10.1155/2011/486756

**Published:** 2011-12-10

**Authors:** Elke R. Ahlmann, Yanling Ma, Vonny Tunru-Dinh

**Affiliations:** ^1^Department of Orthopaedics, Keck School of Medicine, University of Southern California + Los Angeles County Medical Center, 1200 North State Street, GNH 3900, Los Angeles, CA 90033, USA; ^2^Department of Pathology, Keck School of Medicine, University of Southern California + Los Angeles County Medical Center, 1200 North State Street, CT A7A, Los Angeles, CA 90033, USA

## Abstract

Gorham's disease is a rare disorder involving the proliferation of endothelial channels resulting in resorption and disappearance of bone. An unusual case of polyostotic Gorham's disease affecting the scapula, humerus, radius, and ulna in a 39-year-old woman is described. The patient had extensive disease spreading across both the glenohumeral and humeroulnar joints. This is the first report of Gorham's disease spreading across multiple joints in the upper extremity.

## 1. Introduction

Massive spontaneous osteolysis of bone was first described by Gorham et al. [1] in 1954 and later Gorham and Stout [2] in 1955 in which the first 24 cases of this disease were presented. Since then, multiple names have been given to this condition including vanishing bone disease, phantom bone, hemangiomatosis, lymphangiomatosis, and Gorham-Stout syndrome, but it is best known as Gorham's disease [3].

 It is a very rare disorder characterized by abnormal proliferation of thin-walled endothelial-lined channels of vascular or lymphatic origin and an increased number of osteoclasts resulting in progressive resorption of bone [2]. The precise etiology remains largely unknown, and the exact mechanism of bone resorption is unclear. It has a predilection for bones developing by intramembranous ossification and most commonly involves the maxillofacial bones, scapula, clavicle, vertebrae, proximal end of humerus and femur, ribs, ilium, ischium, and sacrum in decreasing order of frequency [3]. The process is usually monostotic, but occasionally can be polyostotic in character [4–6].

To date, approximately, 200 cases of Gorham's disease have been described in the world literature of which approximately 40 have involved the upper extremity [7]. The great majority of these cases describe single bone involvement; however, the spread of disease to adjacent bones has been reported, especially in the upper and lower extremities [2, 8–11]. There have been six case reports of spread from the scapula to the clavicle [1, 10, 12, 13], four reports of spread across the glenohumeral joint from the scapula to the proximal humerus [2, 8], and three cases of involvement of both the forearm and carpal bones [2, 11]. Only one previous case of involvement of the radius and ulna with spread to the distal humerus has been reported [9].

This paper presents an unusual case of polyostotic Gorham's disease affecting the scapula, humerus, radius, and ulna and, to our knowledge, is the first report of this disease spreading across multiple joints in an adult.

## 2. Case Presentation

**Figure 1:**
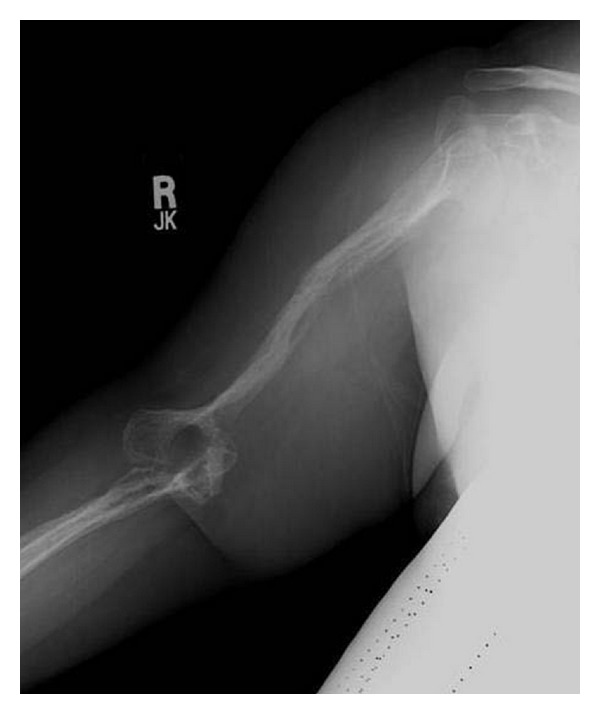
Plain radiographs of the upper extremity taken 8 years earlier revealed a destructive lytic process involving the humerus, radius, and ulna with pathologic midshaft humerus fracture and chronic dislocation of the humeroulnar joint.

A 39-year-old female initially presented to an emergency room 8 years ago complaining of right arm pain after a fall. X-rays taken at that time revealed a destructive lytic process involving the humerus, radius, and ulna with chronic dislocation of the humeroulnar joint (Figure 1). She was referred to an orthopaedic surgeon for evaluation but declined to seek any further medical care for her arm until recently when she presented to our office complaining of an 8-year history of mild right upper extremity pain centered primarily about the elbow. Her symptoms had gradually increased over the past year to the point that she was severely incapacitated and unable to use her right arm. She denied any history of trauma, fevers, or prior surgeries. She had no significant contributory medical or family history.

On physical exam, the skin of the right upper extremity had no cutaneous lesions and there was no sign of infection. Her extremity was diffusely tender and mildly swollen from the shoulder to the wrist. Range of motion of the shoulder, elbow, and wrist was very limited and painful. She had globally decreased motor function and sensation of the hand; however, the vascular examination was normal.

Laboratory studies including complete blood count, complete metabolic panel, alkaline phosphatase, C-reactive protein, erythrocyte sedimentation rate, and serum and urine protein electrophoresis were all within normal limits.

**Figure 2:**
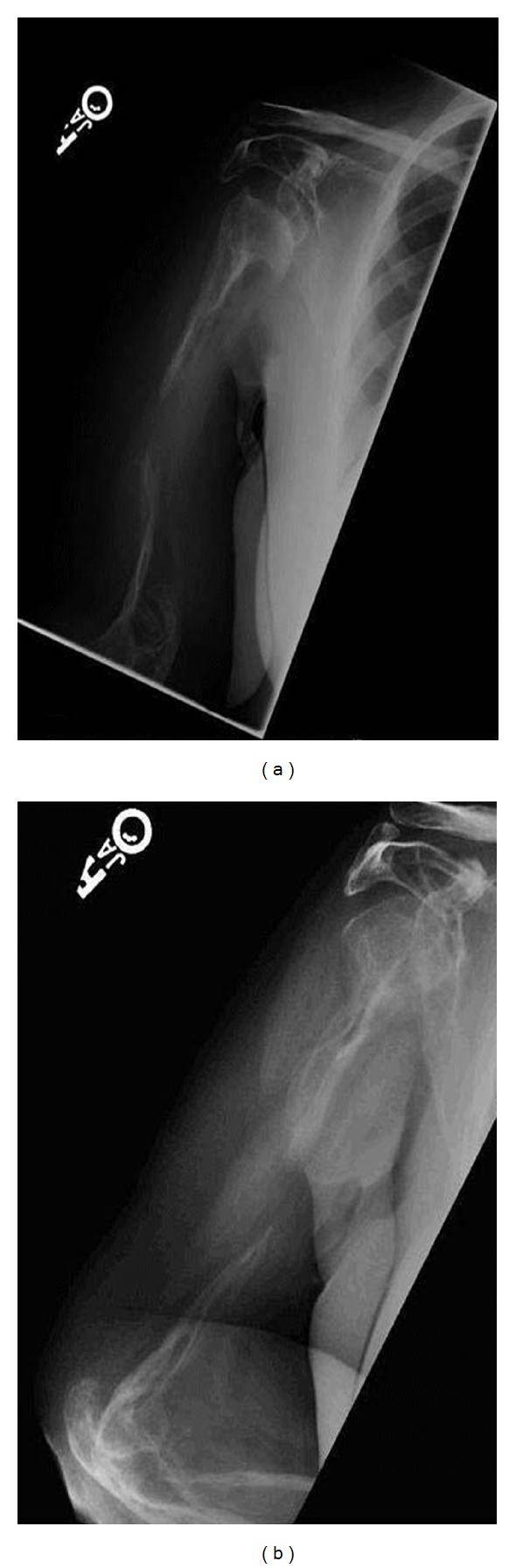
Anteroposterior and lateral plain radiographs of the humerus with diffuse osteopenia and extensive radiolucent foci in the intramedullary and subcortical regions of the bone, and disappearance of the midshaft humerus with tapering of the bone ends. Multiple lytic lesions of the scapula are also visible.

**Figure 3:**
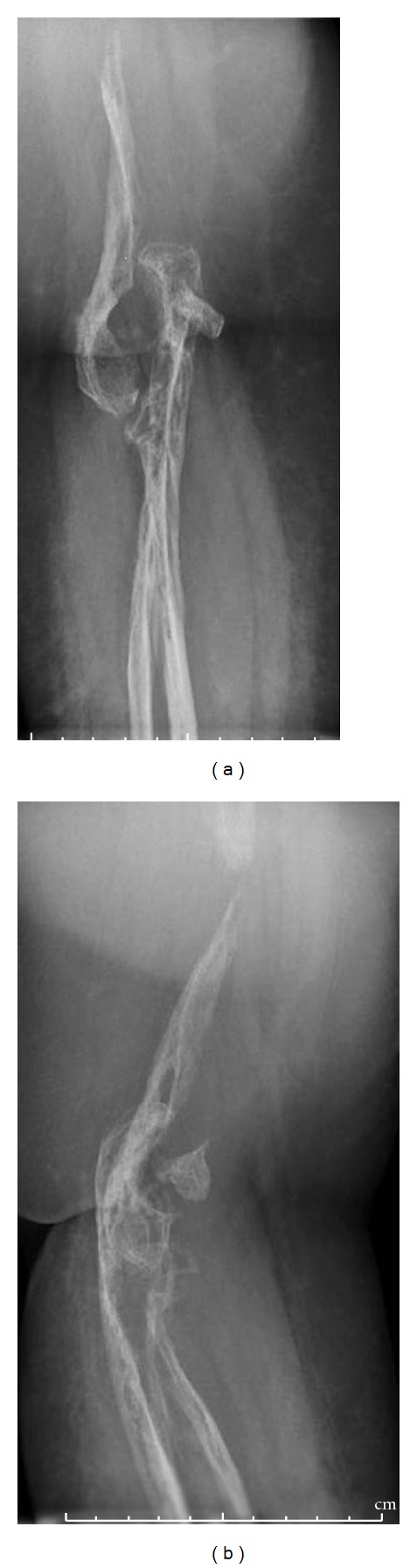
Anteroposterior and lateral plain radiographs of the elbow showing dislocation of the humeroulnar joint and fragmentation and disappearance of the distal humerus.

**Figure 4:**
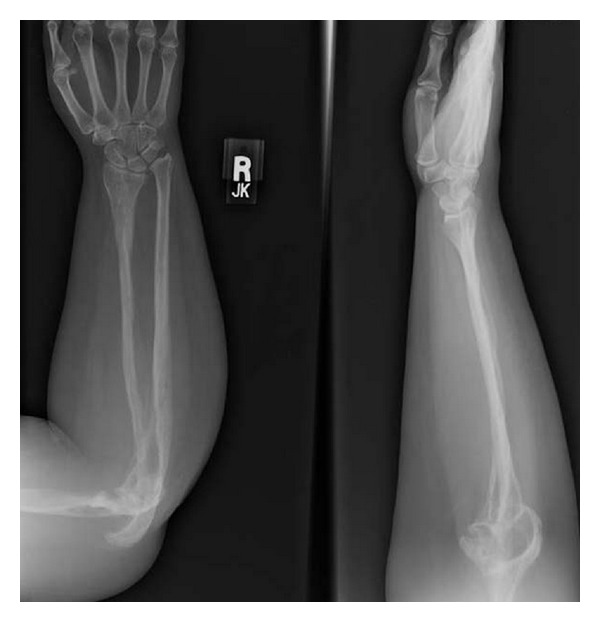
Anteroposterior and lateral plain radiographs of the forearm with multiple subcortical radiolucent foci of both the radius and ulna and subluxation of the distal radioulnar joint.

Plain radiographs of the right upper extremity revealed diffuse osteopenia with extensive radiolucent foci in the intramedullary and subcortical regions of the scapula, humerus, radius, and ulna. She had multiple lytic lesions of the scapula and disappearance of a section of the midshaft of the humerus with tapering of the ends of the bony remnants (Figure 2). Her distal humerus was partially fragmented and dissolved with dislocation of the ulnohumeral joint (Figure 3). Both the radius and ulna were involved with multiple radiolucent foci and subluxation of the distal radioulnar joint (Figure 4). Radiographs of the remainder of her skeleton revealed no abnormalities.

**Figure 5:**
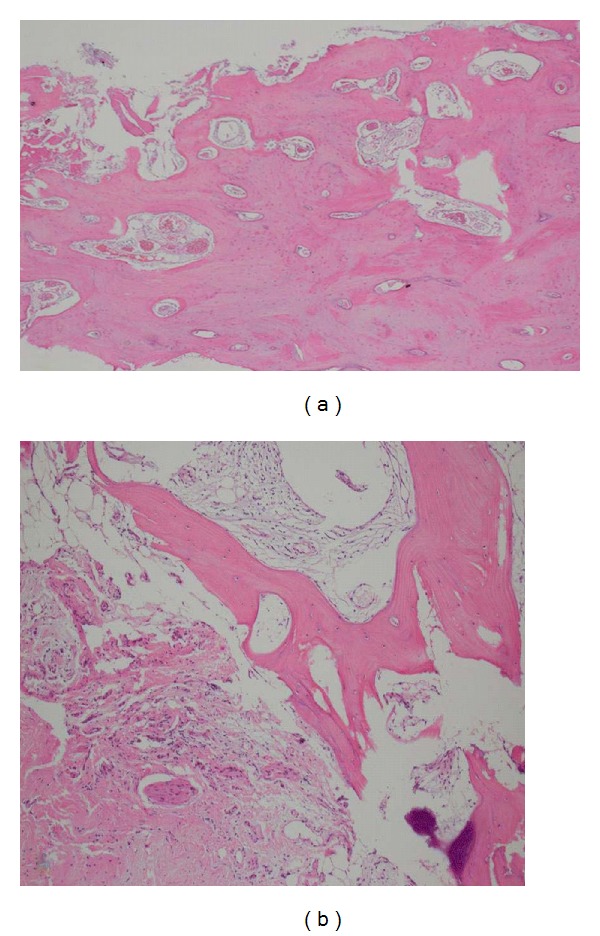
Photomicrograph from the proximal humerus biopsy shows (a) an area of trabecular bone that has been replaced with new vascular channels (hemangiomatous) as well as (b) fibrous tissue replacing the absorbed bone without evidence of regeneration of the bone matrix (hematoxylin & eosin stain, original magnification x100).

An open bone biopsy of the midshaft of the humerus was performed (Figure 5). Histopathologic findings revealed areas of trabecular bone with increased vascular proliferation (hemangiomatous) as well as areas in which fibrous tissue had replaced the absorbed bone without regeneration of new bone matrix. There was no evidence of malignancy or atypical cells.

Both the radiographic and histologic findings were consistent with Gorham's disease, and the patient was started on a course of oral bisphosphonates (alendronate 70 mg once weekly). She completed external beam radiation of 40 Gray (Gy) in 2 Gy fractions. At her latest followup one year after beginning treatment, she has had substantial improvement of her pain although her radiographs remained unchanged. Surgical intervention was not indicated due to the extensive nature of the disease.

## 3. Discussion

Disappearing bone disease or Gorham's disease is a rare disorder characterized by osteolysis with associated proliferation of vascular or lymphatic channels within bone and the surrounding soft tissues [2]. Most cases occur in patients less than 40 years of age and there is no evidence of genetic transmission [3]. The bones most commonly affected are the scapula, clavicle, humerus, pelvis, femur, ribs, skull, maxillofacial bones, mandible, vertebral bodies, hand, and foot [7]. Disease involving the ribs, vertebrae, and shoulder may lead to the development of chylothorax by lymphangiectatic invasion of the thoracic duct or mediastinal extension of the disease into the plural cavity. Without surgical treatment, patients with chylothorax have a very high mortality of greater than 50% [8, 14]. The natural history of Gorham's disease is unpredictable. Spontaneous resolution of osteolysis and regeneration of bone have been reported [15–17], while others have described progression of the disease to the point of disappearance of the entire osseous structure [2]; however, the majority of cases experience spontaneous arrest of the disease. The spread of bone destruction across single joints has been described, mainly in the upper extremity [2, 8, 9, 15]. In the present case, involvement of disease was seen across both the glenohumeral and ulnohumeral joints.

The etiology of Gorham's disease remains largely unclear. Gorham and Stout attributed the bone resorption to progressive replacement of bone by hemangiomatosis or lymphangiomatosis as a result of hyperemia, changes in local pH, and a traumatic “trigger” which stimulates the production of vascular granulation tissue [2]. Some have suggested that there are two stages of Gorham's disease, the first of which is characterized by vascular proliferation in connective tissue which is consistent with hemangiomatosis, followed by a second stage in which fibrous tissue replaces the absorbed bone without regeneration of the bone matrix [18, 19]. Others have suggested that the osteolysis is due to enhanced osteoclast activity with a possible role of interleukin-6 (IL-6) [20, 21]. There has also been evidence to support that cytochemical factors may play a role in activating osteoclasts and causing an imbalance in the process of bone formation and resorption [22, 23]. The exact etiology however remains unknown.

The diagnosis of Gorham's disease is based on clinical and radiologic features of bone loss with histological evidence of angiomatous tumor [3]. Patients may present with sudden onset of pain and swelling, while others have a more insidious onset. Pathologic fracture may be the first presentation of the disease. Most patients develop some degree of soft-tissue atrophy associated with limb disuse, weakness, and decreased range of motion [2, 9, 15]. Other underlying causes of bone destruction such as malignancy, infection, inflammatory, or endocrine disorders must be ruled out with the appropriate radiographic studies and blood tests prior to making the diagnosis of Gorham's disease. The standard laboratory blood tests are usually within normal limits and are generally not helpful in making the diagnosis [3]. A variety of imaging techniques have been used such as plain radiographs, computed tomography scans (CT), magnetic resonance imaging (MRI), and radioisotope bone scans. Gorham's disease can however be best seen on plain radiographs [3, 24]. During the initial stage of the disease, radiolucent foci appear in the intramedullary and/or subcortical regions. This is typically followed by progressive dissolution, fracture, fragmentation, and disappearance of a portion of the bone with tapering of the remaining bone ends [24, 25]. The disease may extend across joints which offers no protection from spread of the disease resulting in regional osseous destruction, commonly around the shoulder or hip [24]. The extent of bone destruction generally increases over several years and may eventually either stabilize or regress [26]. The present case is an example of the progression of disappearing bone disease as the initial radiographs taken 8 years earlier revealed a nondisplaced pathologic midshaft humerus fracture with radiographic evidence of subcortical radiolucent foci suggesting Gorham's disease. When she presented to us, her disease had progressed to the point of complete destruction of the midshaft of the humerus with tapering of the bone ends fitting the classic description of resembling a “licked candy stick” [19, 24, 25].

Once a case is clinically suspicious for Gorham's disease, biopsy of the lesion should be undertaken for definitive diagnosis. On gross examination, the cortex of the involved bone appears thinned and the marrow is replaced with fibrous tissue. The cortex may often appear red, suggesting increased vascularity. On microscopic findings, most have reported vascular proliferation (lymphangiomatous/hemangiomatous) involving bone and the surrounding connective tissue. Osteoclastic activity is generally not prominent, and there should be an absence of atypical cells. In some cases, the microscopic appearance is unremarkable [2, 27]. In the current case, we did see replacement of the trabecular bone with hemangiomatous vascular proliferation and lack of regeneration of bone matrix consistent with Gorham's disease.

Because of the rarity of this disease, the ideal treatment remains uncertain. Therapy has generally centered on surgery [3, 9, 28], radiation [12, 29], antiosteoclastic medication (bisphosphonates) [3, 30], and angiogenesis inhibitors [21, 31]. Surgical options include resection of the lesion and reconstruction with either a bone graft and/or prosthesis [3, 9, 28]. This is typically attempted in monostotic or localized disease in which the entire lesion can be removed. There have however been many complications reported with the use of bone grafts in these patients including fragmentation and dissolution of the graft from erosion of lymphangiomatous tissue resulting in a low success rate [12, 26, 32, 33]. For the present case, surgical treatment was not a feasible option as her disease was extensive, involving several joints and the entirety of multiple bones. Radiation therapy at doses of 40–45 Gy in 2 Gy fractions has been used successfully to result in a good outcome with few complications in many patients, although the main effect has been pain relief and prevention of further bone loss [3, 12, 29]. Regrowth of bone after radiation is unusual [15]. Medical management has centered around the use of bisphosphonates for their antiosteoclastic properties and alpha-2b interferon which acts to prevent angiogenesis and the production of IL-6 [17, 21, 30, 31]. Both treatments have had only variable amounts of success.

In summary, Gorham's disease is a rare entity which can cause extensive destruction of osseous structures and lead to severe disability. There are multiple theories as to the etiology of this disease but none have been definitively proven. The treatment has not yet been fully defined but currently centers around a combination of surgery, radiation, and medical management.

## References

[1] Gorham LW, Wright AW, Shultz HH, Maxon FC (1954). Disappearing bones: a rare form of massive osteolysis. Report of two cases, one with autopsy findings. *The American Journal of Medicine*.

[2] Gorham LW, Stout AP (1955). Massive osteolysis (acute spontaneous absorption of bone, phantom bone, disappearing bone): its relation to hemangiomatosis. *Journal of Bone and Joint Surgery. American*.

[3] Patel DV (2005). Gorham’s disease or massive osteolysis. *Clinical Medicine &amp; Research*.

[4] Scialpi L, Servedio M, Moretti B, Solarino ME, Guglielmo D, Solarino G (2004). Gorham’s disease: a rare case of multicentric localization. *La Chirurgia Degli Organi di Movimento*.

[5] Tauro B (1992). Multicentric Gorham’s disease. *Journal of Bone and Joint Surgery. British*.

[6] Tyler T, Rosenbaum HD (1976). Idiopathic multicentric osteolysis. *American Journal of Roentgenology*.

[7] Papadakis SA, Khaldi L, Babourda EC, Papadakis S, Mitsitsikas T, Sapkas G (2008). Vanishing bone disease: review and case reports. *Orthopedics*.

[8] Tie MLH, Poland GA, Rosenow EC (1994). Chylothorax in Gorham’s syndrome: a common complication of a rare disease. *Chest*.

[9] Rubel IF, Carrer A, Gilles JJ, Howard R, Cohen G (2008). Progressive Gorham disease of the forearm. *Orthopedics*.

[10] Glass-Royal M, Stull MA (1990). Musculoskeletal case of the day. Gorham syndrome of the right clavicle and scapula. *American Journal of Roentgenology*.

[11] Branch HE (1945). Acute spontaneous absorption of bone. Report of a case involving a clavicle and scapula. *Journal of Bone and Joint Surgery. American*.

[12] Dunbar SF, Rosenberg A, Mankin H, Rosenthal D, Suit HD (1993). Gorham’s massive osteolysis: the role of radiation therapy and a review of the literature. *International Journal of Radiation Oncology Biology Physics*.

[13] Damron TA, Brodke DS, Heiner JP, Swan JS, DeSouky S (1993). Case report 803: Gorham’s disease (Gorham-Stout) syndrome of scapula. *Skeletal Radiology*.

[14] Lee WS, Kim SH, Kim I (2002). Chylothorax in Gorham’s disease. *Journal of Korean Medical Science*.

[15] Campbell J, Almond HGA, Johnson R (1975). Massive osteolysis of the humerus with spontaneous recovery. Report of a case. *Journal of Bone and Joint Surgery. British*.

[16] Kamath RP, Chandran P, Malek S, Mohsen AMMA (2007). Rapid, spontaneously resolving osteolysis of the hand. *Orthopedics*.

[17] Somoza Argibay I, Díaz González M, Martínez Martínez L, Ros Mar Z, López-Gutiérrez JC (2003). Heterogenicity of Gorham-Stout syndrome: association with lymphatic and venous malformations. *Anales de Pediatria*.

[18] Heffez L, Doku HC, Carter BL, Feeney JE (1983). Perspectives on massive osteolysis. Report of a case and review of the literature. *Oral Surgery Oral Medicine and Oral Pathology*.

[19] Johnson PM, McClure JG (1958). Observations on massive osteolysis; a review of the literature and report of a case. *Radiology*.

[20] Moller G, Priemel M, Amling M, Werner M, Kuhlmey AS, Delling G (1999). The Gorham-Stout syndrome (Gorham’s massive osteolysis). A report of six cases with histopathological findings. *Journal of Bone and Joint Surgery. British*.

[21] Devlin RD, Bone HG, Roodman GD (1996). Interleukin-6: a potential mediator of the massive osteolysis in patients with Gorham-Stout disease. *Journal of Clinical Endocrinology and Metabolism*.

[22] Dickson GR, Mollan RAB, Carr KE (1987). Cytochemical localization of alkaline and acid phosphatase in human vanishing bone disease. *Histochemistry*.

[23] Hirayama T, Sabokbar A, Itonaga I, Watt-Smith S, Athanasou NA (2001). Cellular and humoral mechanisms of osteoclast formation and bone resorption in Gorham-Stout disease. *Journal of Pathology*.

[24] Resnick D, Resnick D (2002). Osteolysis and chondrolysis. *Diagnosis of Bone and Joint Disorders*.

[25] Torg JS, Steel HH (1969). Sequential roentgenographic changes occurring in massive osteolysis. *Journal of Bone and Joint Surgery. American*.

[26] Cannon SR (1986). Massive osteolysis. A review of seven cases. *Journal of Bone and Joint Surgery. British*.

[27] Unni KK, Inwards CY, Bridge JA, Kindblom L, Wold LE, Unni KK (2005). Vascular tumors. *Tumors of the Bones and Joints*.

[28] Poirier H (1968). Massive osteolysis of the humerus treated by resection and prosthetic replacement. *Journal of Bone and Joint Surgery. British*.

[29] Fontanesi J (2003). Radiation therapy in the treatment of Gorham disease. *Journal of Pediatric Hematology/Oncology*.

[30] Avelar RL, Martins VB, Antunes AA, de Oliveira Neto PJ, de Souza Andrade ES (2010). Use of zoledronic acid in the treatment of Gorham’s disease. *International Journal of Pediatric Otorhinolaryngology*.

[31] Hagberg H, Lamberg K, Åström G (1997). *α*-2b interferon and oral clodronate for Gorham’s disease. *The Lancet*.

[32] Heyden G, Kindblom andMoeller Nielsen LGJ (1977). Disappearing bone disease. A clinical and histological study. *Journal of Bone and Joint Surgery. American*.

[33] Mendez AA, Keret D, Robertson W, MacEwen GD (1989). Massive osteolysis of the femur (Gorham’s disease): a case report and review of the literature. *Journal of Pediatric Orthopaedics*.

